# Diversity Outbred: A New Generation of Mouse Model

**DOI:** 10.1289/ehp.123-A64

**Published:** 2015-03-01

**Authors:** Charles W. Schmidt

**Affiliations:** Charles W. Schmidt, MS, an award-winning science writer from Portland, ME, has written for *Discover Magazine*, *Science*, and *Nature Medicine*.

Most of the mice used for testing the toxic effects of chemicals and drugs are genetically inbred with a long history in the laboratory.^1^ But toxicologists are increasingly turning to newer mouse models that more accurately mimic the genetic diversity of the human population. Investigators with the National Toxicology Program (NTP) at the National Institute of Environmental Health Sciences have now reported that one such model—the Diversity Outbred (DO) mouse model—varies widely in its susceptibly to benzene, a known cause of human leukemia.^2^ The results demonstrate the model’s improved capacity for identifying subtle chemical effects and lend further credibility to the use of DO mice in toxicology research and safety assessment, according to lead author John E. French, a toxicologist specializing in toxicogenetics formerly with NTP and now an adjunct professor in the Center for Pharmacogenomics and Individualized Therapy at the University of North Carolina at Chapel Hill.

**Figure:**
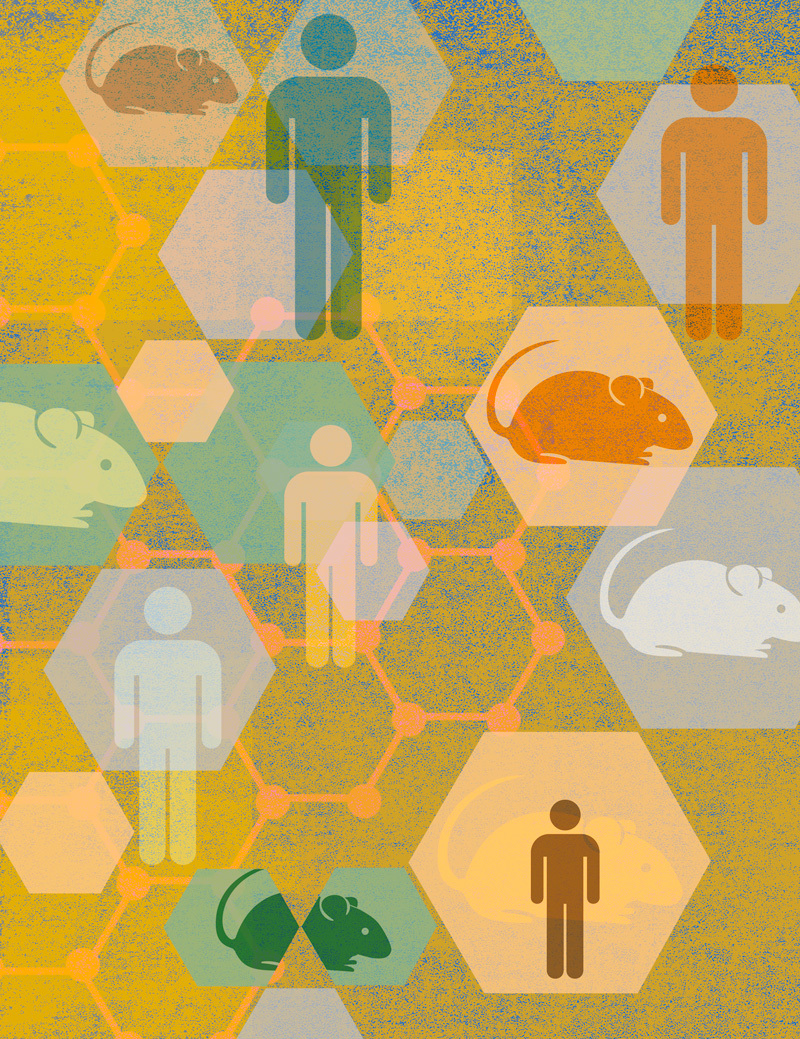
The use of genetically identical mice in toxicology studies can make it tricky to extrapolate findings to people. A new mouse model known as the Diversity Outbred better reflects the genetic diversity of the human population, offering intriguing possibilities for safety assessment. © Roy Scott Design/Illustration

## Proof of Concept

Because toxicity depends in part on how chemicals and genes interact, genetically inbred mice—generated by breeding siblings—tend to respond similarly to the agents tested in a given study. That has certain advantages; for instance, it limits the number of animals needed to detect statistically significant differences in chemical effects. But among other disadvantages, it’s possible that inbred mice might exhibit strain-specific responses with little relevance to the genetically diverse human population, says Kristine Witt, a toxicologist with the NTP.^3^

It’s not unusual for toxicologists to work with outbred mouse strains derived from unrelated pairings. These strains have more varied reactions to chemicals and drugs, but they also vary unpredictably with respect to their own “outbredness.” By contrast, the DO model is maintained under strict randomized breeding conditions designed to ensure that only unrelated mice mate.^4^^,^^5^ Thus, every DO mouse is genetically unique. Moreover, the eight “founders”—the original parental strains of mice from which all subsequent DO generations derive—were fully sequenced,^6^^,^^7^ “and so we can reconstruct the genome of any single DO mouse with a high degree of precision,” says Gary Churchill, a professor at Jackson Laboratories in Bar Harbor, Maine. That ability, Churchill says, facilitates genomewide association studies that aim to pinpoint the genes or alleles that govern a particular trait.

For the new proof-of-concept study,^2^ NTP investigators and their collaborators exposed two independent cohorts of 300 male DO mice each to benzene. This chemical was chosen because its metabolism *in vivo* is well characterized and known to be similar in mice and humans. “The possibility of finding distinct gene associations in the response to benzene exposure, based on the diversity of the metabolic pathways involved, seemed high,” says Witt, a coauthor.

Groups of 75 mice each were exposed to benzene in air at 0, 1, 10, or 100 ppmv for 28 days. Then the investigators looked at peripheral blood and bone marrow samples for evidence of micronuclei (MN). MN arise from chromosomal fragments or whole chromosomes that fail to incorporate into daughter nuclei during cell division, and their numbers are known to increase dose-dependently with benzene exposure.

MN counts in peripheral blood were significantly different in mice with the highest exposure compared with unexposed animals, but were similar to unexposed mice for those animals with lower exposures. MN counts in bone marrow, however, differed from nonexposed controls at every dose level.^2^ “We can’t sample the bone marrow in exposed humans, but these results suggest that changes in blood may not reflect bone marrow toxicity among the most sensitive individuals,” French says.

Like DO mice, humans differ in their susceptibility to benzene, with some showing evidence of blood toxicity at exposure levels below the federal occupational standard.^8^^,^^9^^,^^10^^,^^11^^,^^12^ Importantly, though, the benchmark concentration was an order of magnitude lower than the concentration estimated in earlier studies with inbred B6C3F1 hybrid mice, which have been used routinely by the NTP since the 1970s and are still in widespread use today.^2^ The benchmark concentration is the concentration associated with a small but measurable biological response—in this case, at most a 10% increase in micronucleation compared with nonexposed animals.

When the investigators repeated the same experiment four months later, they got the same results. As before, individual DO mice varied in their response to benzene, but the cohorts’ overall variation was very similar to that seen in the first study.^2^

“There was no statistical difference between the data sets,” Witt says. “All the exposed mice were each genetically different from the others, with different coat colors and temperaments—just like humans. But even so, our results were reproducible. This observation was crucial for convincing the toxicology community that DO mice can be a useful tool.” If the two data sets had been wildly different, she says, then the DO model would not be seen as reliable for chemical testing.

By performing linkage analyses on the mouse genomes, the investigators were able to home in on genes that confer resistance to benzene toxicity—most likely a group of two sulfotransferases located on chromosome 10 that modify and eliminate benzene metabolites.^2^ Witt says the sulfotransferases could modify benzene metabolites in ways that limit their ability to reach or harm bone marrow, the source of the blood stem cells that can give rise to benzene-induced leukemia. Humans have analogous sulfotransferases that are known to have similar activity. She says, “This illustrates how genetic results from toxicity studies in DO mice can guide us toward related genes in humans for further study and can help elucidate underlying mechanisms of action leading to toxicity and disease.”

Michael DeVito, acting chief of the NTP Laboratory, says DO mice could help toxicologists ensure that they don’t miss a potentially significant human end point. He says the NTP is now working to better characterize the animals with respect to baseline differences in serum chemistry, organ weights, reproductive capacity, and other measures, with the anticipation that the model may eventually be incorporated into NTP testing protocols. “The more of these studies we do, the better will be our understanding of the normal population variation,” French says.

## The Founders

The DO mice were created during the last decade from a predecessor model called the Collaborative Cross (CC).^13^^,^^14^^,^^15^ Efforts to create the CC date back to 2002.^16^ David Threadgill, a geneticist and professor at Texas A&M University, says scientists at the time had become increasingly aware that genetic background can dictate phenotype in toxicology. Worried that they might be missing important human end points by relying on established inbred strains in research, Threadgill and other scientists created the Complex Traits Consortium (CTC) with a mission, he says, “to reinvent the mouse model so that it would contain genetic variability on the scale of what exists in humans.”

To accomplish that mission, the CTC crossbred eight founder strains from the three major laboratory and wild subspecies of *Mus musculus,* otherwise known as the house mouse. Analyses confirmed that the eight strains captured 90% of the genetic variation known to exist in *M. musculus*, and that the variation was randomly distributed across the genome.^13^^,^^17^ The eight strains were crossbred using a “funnel” design that sequentially narrowed generational matings. Eventually, siblings were mated to generate inbred strains, “each with a random sampling of the genetic variation that was initially present in the founders,” Threadgill explains.

According to Churchill, inbred CC strains are defined on the basis of two criteria: Their genomes must contain DNA from at least six of the eight founders, and they must display 98% homozygosity, meaning the copies of each gene inherited from the mother are identical to the copies inherited from the father.

To maximize access to the founders’ genomic diversity, scientists experiment with as many different CC strains as they can, DeVito says. This approach was illustrated in a landmark 2014 paper by researchers who worked with 47 CC strains and found that they exhibited varying reactions to the Ebola virus, just as humans do.^18^ Traditional inbred mouse models don’t develop the human-like symptoms of Ebola hemorrhagic fever, which include delayed blood coagulation, intravascular blood clots, and potentially death from shock. But according to this widely reported paper, some CC strains do exhibit these symptoms, with lethality in the animals dependent on genetic background—susceptible animals showed 10- to 100-fold increases in the expression of genes that induce inflammation, cell death, and vascular leakage. By contrast, genes that limit vascular leakage, possibly by facilitating repair of blood vessels, were upregulated in resistant mice. Genetic factors may therefore play a significant role in determining human survival of infection with the Ebola virus, the authors speculated.^18^

According to Churchill, the CTC’s initial goal was to breed up to 1,000 CC strains. Yet that proved unfeasible because so many of the strains died out over time. “We ran into fertility problems,” Churchill explains. “After about five generations, ninety percent of the strains would stop producing pups.” That was, to some extent, a predictable setback, Churchill adds, given that inbred animals often suffer from health problems and poor reproductive capacity.

Still, some CC strains bred vigorously, and the panel now comprises roughly 200 recombinant inbred strains, of which 90 currently are publicly available; as the remaining CC strains are inbred, they will be released to the public, Threadgill says. Those strains will ideally contain the genetic variation researchers need to map the genes they’re looking for in a given study—for instance, genetic traits that might predict outcomes among Ebola patients. “But luck also plays into the game,” Churchill says. “If you go through all the available CC strains and you still come up empty-handed, then you’ve hit a wall.”

## Developing the DO Model

That limitation is what galvanized scientists to develop the DO mouse model in 2009.^4^^,^^19^ To generate DO mice, scientists randomly breed across the different CC strains. Random mating minimizes the potential for genetic drift, or the loss of genetic variety in the population, Threadgill explains. Thus, genetic diversity is broken out into finer and finer scales, and according to Threadgill, this allows for far more resolution in genetic analysis than is achievable in CC strains with a fixed genetic structure.

Upon finding the genes that govern a particular trait in DO mice, researchers can then check to see if those genes are also present in a given CC line. This is important because it’s impossible to reproduce genetically identical DO cohorts. Since all the animals in a given cohort are genetically unique, researchers have no way of knowing if genes of interest found in one group of DO mice will also be present in another group. But if those same genes can be subsequently identified in a CC strain, then that strain can be continually replenished for ongoing research. In that sense, Churchill says, the DO and CC models complement each other—researchers can hunt for genes in DO animals, and then go on to study the genes they find in a renewable pool of CC mice.

Still, DO mice pose a fundamental challenge to research and testing: Because it’s impossible to know which animals have the genes and allelic variants of interest, researchers by necessity have to search for them in as many animals as possible. According to Threadgill, the specific number depends on the complexity of the genetic pathways involved. “If you’ve got a simple pathway with just three to four genes controlling a given trait, you can get by with fewer animals,” he says. “That’s not true for highly variable traits controlled by lots of different genes, however.”

DeVito acknowledges that sample size and statistical power requirements with DO mice are open questions at the NTP. To gain a better understanding of their physiology, DeVito and his colleagues recently launched a pilot study. They put 75 DO mice on a high-fat diet, and then compared changes in serum chemistry, histology, organ and body weight, and other end points with those of control DO animals fed normal diets. Unpublished results showed that individual animals from either group differed little with respect to these end points, except for sperm counts, which varied tremendously in both the control and high-fat groups for unknown reasons.

“It’s not like we had a few extreme outliers,” DeVito says. “Instead, the sperm counts rose gradually among the animals, with a seventyfold difference between the lowest and the highest values.”

For context, DeVito points out that B6C3F1 mice normally have no more than a twofold difference in sperm counts. The fact that the counts vary so widely in DO mice presents research difficulties, especially for studies of male reproductive toxicants. Instead of using 10–20 animals per treatment group, which is what NTP guidelines recommend in studies with inbred strains, scientists would probably need to use hundreds of DO mice to pick up subtle reproductive effects that could be distinguished from results in untreated controls, according to DeVito.

“Traditional study designs will not have the same statistical power in the DO as they do in more typical inbred strains,” DeVito says. “We need to better understand the variability in the untreated DO mouse for any end point so that we can appropriately design a study for this model.”

All that said, DO and CC mice both offer promising opportunities for chemical risk assessment, says Weihsueh Chiu, a professor at the College of Veterinary Medicine and Biomedical Sciences at Texas A&M University. According to Chiu, DO and CC mice offer three fundamental benefits: 1) they improve hazard identification by allowing scientists to pick up toxic effects that might not be evident in a resistant inbred strain; 2) they improve dose–response assessment by modeling human genetic diversity; and 3) they improve mechanistic understanding through techniques such as genomewide association studies to identify potential pathways governing toxic resistance or susceptibility to toxicity.

But Chiu acknowledges that the benefits of genetic variability come with a tradeoff. DO and CC mice are more expensive than other laboratory mice, Chiu notes, and costs must be balanced with statistical power requirements, echoing the study design issues raised by DeVito.

“The essential question is this: In what cases do the benefits in terms of hazard identification, dose response, or mechanistic understanding justify the additional costs of using DO or CC mice?” Chiu asks. “Right now, we have proof of concept that they can be useful. We’re in a development and refinement stage, and I’m confident that in the process, we can figure out how best to use them to support our ultimate goal of protecting public health.”
